# From structure to mechanism—understanding initiation of DNA replication

**DOI:** 10.1101/gad.298232.117

**Published:** 2017-06-01

**Authors:** Alberto Riera, Marta Barbon, Yasunori Noguchi, L. Maximilian Reuter, Sarah Schneider, Christian Speck

**Affiliations:** 1 DNA Replication Group, Institute of Clinical Sciences (ICS), Faculty of Medicine, Imperial College London, London W12 0NN, United Kingdom;; 2 Medical Research Council (MRC) London Institute of Medical Sciences (LMS), London W12 0NN, United Kingdom

**Keywords:** MCM2–7, DNA replication, pre-RC, CMG, replisome, cryo-EM

## Abstract

DNA replication results in the doubling of the genome prior to cell division. This process requires the assembly of 50 or more protein factors into a replication fork. Here, we review recent structural and biochemical insights that start to explain how specific proteins recognize DNA replication origins, load the replicative helicase on DNA, unwind DNA, synthesize new DNA strands, and reassemble chromatin. We focus on the minichromosome maintenance (MCM2–7) proteins, which form the core of the eukaryotic replication fork, as this complex undergoes major structural rearrangements in order to engage with DNA, regulate its DNA-unwinding activity, and maintain genome stability.

## Abstract (precis)

In this Review, Riera et al. review recent structural and biochemical insights that start to explain how specific proteins recognize DNA replication origins, load the replicative helicase on DNA, unwind DNA, synthesize new DNA strands, and reassemble chromatin.

Initiation of DNA replication is a multistep reaction that is carefully choreographed to promote replication fork assembly and regulated firing of replication origins (Costa et al. 2013; Riera et al. 2014; Tognetti et al. 2015; Bell and Labib 2016; Deegan and Diffley 2016; O'Donnell and Li 2016; Pellegrini and Costa 2016; Riera and Speck 2016; Bleichert et al. 2017). Moreover, this process is highly regulated in order to coordinate DNA synthesis with the cell cycle and the energy status of the cell. Checkpoints monitor the pathway and can halt DNA synthesis to overcome problems and safeguard the genome from damage (Alexander and Orr-Weaver 2016). In vivo analysis has identified most key players and addressed many regulatory principles, but many crucial mechanisms remain unknown. The recent reconstitution of budding yeast DNA replication using purified proteins now offers the chance for a detailed mechanistic and structural analysis of DNA replication initiation and DNA synthesis, which will help to advance the entire field (Yeeles et al. 2015, 2017; Devbhandari et al. 2017).

## Initiation of DNA replication

**Figure 1.**
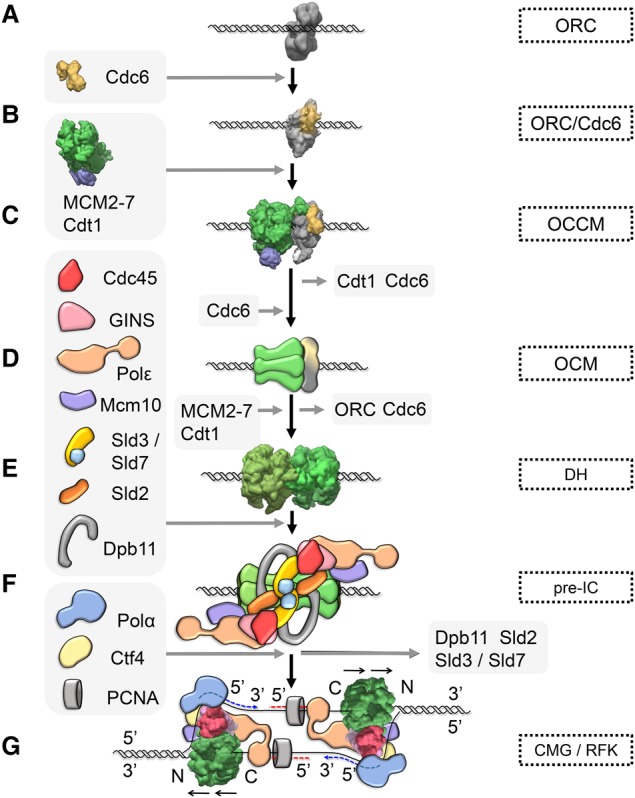
Eukaryotic initiation of DNA replication. Relevant complexes that have been characterized by electron microscopy are shown in surface view. (*A*) ORC is chromatin bound throughout the cell cycle (Electron Microscopy Data Bank [EMDB]: 1156). (*B*) ORC/Cdc6 is the landing platform MCM2–7/Cdt1 (EMDB: 5381). (*C*) The association of MCM2–7/Cdt1 (EMDB: 6671) with ORC/Cdc6 results in the OCCM (EMDB: 8540) formation with the dsDNA inserted into MCM2–7 hexamer. (*D*) Cdt1 and Cdc6 are released from the OCCM in an ATP hydrolysis-dependent reaction, and, upon recruitment of another Cdc6, the OCM the complex is formed. The OCM is an essential intermediate in the loading reaction and is responsible for the recruitment of a second MCM2–7/Cdt1 heptamer, although the details of the reaction are currently not known. (*E*) The final product of the loading reaction is a MCM2–7 DH embracing dsDNA (EMDB: 6338). This inactive complex is a stable DNA replication intermediate, which becomes activated only in S phase. (*F*) Preinitiation of DNA replication in S phase relies on Dbf4-dependent kinase (DDK)-dependent phosphorylation of the DH and a plethora of factors that interact with the DH. One of the landmarks of preinitiation complex formation is the binding of Cdc45 and GINS (from the Japanese go-ichi-ni-san, meaning 5-1-2-3, after the four related subunits of the complex: Sld5, Psf1, Psf2, and Psf3) to MCM2–7, resulting in formation of the replicative helicase: the Cdc45/MCM2–7/GINS (CMG) complex. (*G*) During the DNA-unwinding process, the CMG (EMDB: 8518) associates with both polymerases ε and α into a replication fork (RFK) to synthesize the leading and lagging strands. The helicase is propelled by the C-terminal AAA^+^ motor domains, and the unwinding takes place on the N-terminal face.

The genomic sites where DNA replication is initiated are known as DNA replication origins (Marahrens and Stillman 1992; Hyrien 2016). In budding yeast, replication origins have conserved DNA sequences and always contain a binding site for the origin recognition complex (ORC) (Fig. 1A; Bell and Stillman 1992). This complex consists of six subunits and is organized in a C shape, with DNA being inserted into the central cleft, allowing for multiple protein–DNA contacts (Lee and Bell 1997; Speck et al. 2005; Sun et al. 2012; Yuan et al. 2017). During late M phase of the cell cycle, Cdc6 binding to an ORC/origin DNA complex starts a process of regulated protein assembly that culminates in the formation of the replication fork in S phase (Fig. 1B; Donovan et al. 1997; Rowles and Blow 1997; Weinreich et al. 1999). The ORC/Cdc6 complex contains four ATP-binding proteins: Cdc6, Orc1, Orc4, and Orc5. ATP binding by Orc1 and Cdc6 is required for ORC/Cdc6/DNA complex formation (Weinreich et al. 1999; Gillespie et al. 2001; Klemm and Bell 2001; Speck et al. 2005; Randell et al. 2006; Speck and Stillman 2007). Importantly, this ORC/Cdc6 complex is essential for the recruitment of the Cdt1/minichromosome maintenance 2–7 (MCM2–7) heptamer and the loading of MCM2–7 on dsDNA (Fig. 1C). MCM2–7 is the core of the replicative DNA helicase and consists of six subunits that have a spiral arrangement with a gap at the Mcm2/5 interface (Costa et al. 2011; Ticau et al. 2017; Zhai et al. 2017). During helicase loading, ORC, Cdc6, and Cdt1 insert DNA through the Mcm2/Mcm5 gate into the complex, and the MCM2–7 ring closes partially around dsDNA (Sun et al. 2013; Samel et al. 2014; Zhai et al. 2017). Crucially, the MCM2–7 recruitment process depends on an Mcm6–Cdt1 interaction, which alleviates an autoinhibitory activity of the Mcm6 C terminus (Fernandez-Cid et al. 2013). Upon ORC/Cdc6/Cdt1/MCM2–7 (OCCM) complex formation, ATP hydrolysis results in sequential Cdc6 and Cdt1 release and formation of an ORC/MCM2–7 (OM) intermediate (Fernandez-Cid et al. 2013; Coster et al. 2014; Kang et al. 2014; Ticau et al. 2015). Interestingly, Cdt1 release is associated with a structural change, as it promotes the closure of the MCM2–7 ring (Ticau et al. 2017). Following the recruitment of a second Cdc6, an ORC/Cdc6/MCM2–7 (OCM) complex is formed (Fig. 1D; Fernandez-Cid et al. 2013; Ticau et al. 2015, 2017). In contrast to the OCCM and the OM, the OCM complex is competent to rapidly recruit a second MCM2–7 hexamer, which also occurs in a Cdt1-dependent manner (Evrin et al. 2013, 2014; Fernandez-Cid et al. 2013; Sun et al. 2014; Ticau et al. 2015), resulting in a head-to-head MCM2–7 double hexamer (DH) that encircles dsDNA (Fig. 1E; Evrin et al. 2009; Remus et al. 2009; Gambus et al. 2011). Formation of the DH triggers Cdc6 release followed by simultaneous ORC and Cdt1 release and closure of the second MCM2–7 ring around DNA, resulting in a high-salt-stable complex (Evrin et al. 2009; Remus et al. 2009; Ticau et al. 2015, 2017). DH loading is also termed as prereplication complex (pre-RC) formation or DNA licensing. The large MCM2–7 DH is devoid of ATP hydrolysis (Sun et al. 2014) and DNA-unwinding activities (Evrin et al. 2009; Remus et al. 2009) but can slide on dsDNA in an ATP hydrolysis-independent manner (Evrin et al. 2009; Remus et al. 2009) to distribute DHs to non-origin-containing regions (Gros et al. 2015).

Activation of the MCM2–7 DH, termed preinitiation complex (pre-IC) formation, is a highly complex process and has been intensively studied in budding yeast. It depends on Dbf4-depedent kinase (DDK) Cdc7 and S-phase-specific cyclin-dependent kinase (CDK) and a large number of activation factors, including Sld3, Cdc45, Sld2, Dpb11, GINS (from the Japanese go-ichi-ni-san, meaning 5-1-2-3, after the four related subunits of the complex: Sld5, Psf1, Psf2 and Psf3), polymerase ε, and Mcm10 (Fig. 1F; Heller et al. 2011; Yeeles et al. 2015). During S phase, the MCM2–7 DHs become efficiently phosphorylated by DDK (Sheu and Stillman 2006, 2010; Francis et al. 2009; Sun et al. 2014), which in turn allows Sld3/Sld7 and Cdc45 recruitment to replication origins (Bruck et al. 2015; Herrera et al. 2015; Deegan et al. 2016; Fang et al. 2016). Moreover, CDK phosphorylates its essential targets, Sld2 and Sld3, which then allows these two phospho-proteins to interact with Dpb11 (Tanaka et al. 2007; Zegerman and Diffley 2007). Sld2, Dpb11, GINS, and polymerase ε form a loose complex in the cell (Muramatsu et al. 2010) and bind to Cdc45/Sld3/MCM2–7. Consequently, Sld2, Sld3, and Dpb11 are released, resulting in formation of the Cdc45/MCM2–7/GINS (CMG) complex (Kanemaki and Labib 2006; Heller et al. 2011; Yeeles et al. 2015). Interestingly, Mcm10 binding to the CMG is associated with a structural change in the CMG, rendering the complex high-salt-stable (Looke et al. 2017) and promoting origin firing (Kanke et al. 2012; van Deursen et al. 2012; Watase et al. 2012; Yeeles et al. 2015). The completely assembled CMG complex is highly active in ATP hydrolysis-driven 3′–5′ DNA unwinding, as seen first for the *Drosophila* variant, and forms the center of the eukaryotic DNA replication fork (Fig. 1G; Moyer et al. 2006; Ilves et al. 2010).

Crucially, during helicase activation, the MCM2–7 DH becomes extensively reorganized: Cdc45 and GINS bind to MCM2–7; the complex is split into two individual hexamers, potentially involving Mcm10 (Quan et al. 2015); one DNA strand becomes extruded from each hexamer; and this results in the two CMG complexes encircling ssDNA (Costa et al. 2014; Georgescu et al. 2017). Importantly, the mechanisms that lead to this MCM2–7 reorganization during initiation of DNA replication are largely unknown. DNA polymerases are known to associate with the CMG in part via Ctf4 to form a coupled DNA-unwinding and DNA synthesis assembly (Fig. 1G; Gambus et al. 2006; Simon et al. 2014; O'Donnell and Li 2016). Initial priming of DNA synthesis is carried out by DNA polymerase α, while leading and lagging strand DNA synthesis occurs mainly via polymerase ε (Pursell et al. 2007; Burgers et al. 2016) and polymerase δ (Nick McElhinny et al. 2008), respectively. However, some plasticity exists, with polymerase δ also playing a role during leading strand DNA synthesis, particularly during initiation of DNA replication and under conditions of replicative stress in budding yeast (Pavlov et al. 2001; Devbhandari et al. 2017; Yeeles et al. 2017) and after homologous recombination-dependent fork restart in *Schizosaccharomyces pombe* (Miyabe et al. 2015). Mcm10 acts also during elongation, as it travels with the replisome (Ricke and Bielinsky 2004; Gambus et al. 2006; Pacek et al. 2006), and as a specific mutation in its C terminus results in shorter replication products without affecting origin firing (Looke et al. 2017).

DNA synthesis is initiated at hundreds to thousands of replication origins in order to fully replicate the large eukaryotic genomes (Cvetic and Walter 2005; Mechali 2010). At each origin, one or more MCM2–7 DHs are loaded, but only a minority becomes transformed into active CMGs during S phase; with the remaining MCM2–7 DHs serving as “dormant origins,” which become activated only if a proximal replication fork becomes terminally arrested (Woodward et al. 2006; Ibarra et al. 2008).

## Structural insights into key steps of DNA replication

The last few years have seen rapid progress for structural biology, in part due to the resolution revolution of cryo-electron microscopy (cryo-EM) (Kuhlbrandt 2014; Egelman 2016), and this has led to outstanding biology insights into DNA replication. Crystallography and cryo-EM have generated near-atomic resolution structures of several large protein complexes, including *Drosophila melanogaster* and *Homo sapiens* ORC (Bleichert et al. 2015; Tocilj et al. 2017); *Saccharomyces cerevisiae* MCM2–7 and MCM2–7/Cdt1 (Zhai et al. 2017), OCCM complex (Yuan et al. 2017), and MCM2–7 DH (Li et al. 2015); *S. cerevisiae* and *D. melanogaster* CMG (Abid Ali et al. 2016; Yuan et al. 2016; Georgescu et al. 2017); *S. cerevisiae* Ctf4 trimer (Simon et al. 2014), polymerase ε (Hogg et al. 2014), and polymerase δ (Swan et al. 2009); and *H. sapiens* Mcm2-H3/H4 (Huang et al. 2015; Richet et al. 2015). Here we concentrate on four key complexes involved in initiation of DNA replication; namely, *S. cerevisiae* ORC, OCCM, MCM2–7 DH, and CMG (Fig. 1A,C,E,G).

## Origin recognition

**Figure 2.**
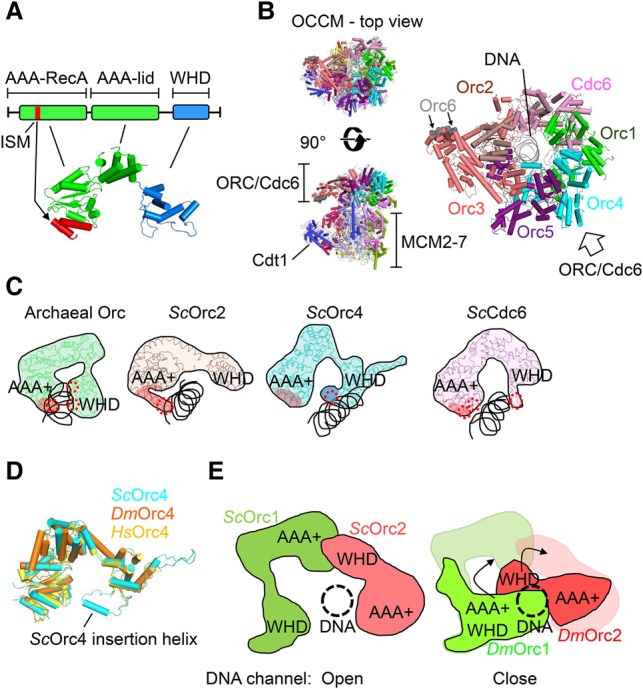
Origin recognition. (*A*) The domain and structural organization of Orc and Cdc6 proteins. The initiator-specific motif region of the AAA-RecA domain is indicated in red. (*B*, *left*) The OCCM structure in top view and side view (Protein Data Bank [PDB]: 5UDB). (*Right*) A top view of the ORC/Cdc6–DNA structure is shown enlarged. (*C*) Archaeal Orc (PDB: 2V1U) and the DNA-binding subunits of *S. cerevisiae* (*Sc*) ORC and Cdc6 are shown (PDB: 5UDB). The DNA-binding regions are indicated by red circles. The Orc4 insertion helix is shown in dark blue. (*D*) An overlay of *S. cerevisiae* Orc4 (PDB: 5UDB), *D. melanogaster* (*Dm*) Orc4 (PDB: 4XGC), and *H. sapiens* (*Hs*) Orc4 (PDB: 5UJM). (*E*) Structural comparison of *D. melanogaster* Orc1–Orc2 with *S. cerevisiae* Orc1–Orc2. The arrows show the rotation of *D. melanogaster* Orc1 AAA^+^ and Orc2 WHD to fit the *S. cerevisiae* Orc1 AAA^+^ and Orc2 WHD positions.

Sequence-specific recognition of DNA replication origins by the *S. cerevisiae* ORC formed the biochemical basis for the discovery of this important DNA replication factor in 1992 (Bell and Stillman 1992), which started a 25-year journey toward the full reconstitution of budding yeast DNA replication (Yeeles et al. 2015, 2017; Devbhandari et al. 2017). The six-subunit Orc1–6 complex is well conserved from yeast to humans, and this homology extends even to archaea, where Orc monomers or dimers function in origin recognition (Li and Stillman 2012). Orc1–5, but not Orc6, have a conserved protein structure consisting of one N-terminal AAA^+^ domain and one C-terminal winged helix domain (WHD) (Fig. 2A; Bleichert et al. 2015; Tocilj et al. 2017; Yuan et al. 2017). Structural analysis showed that *H. sapiens* Orc6 has homology with transcription factor TFIIB (Liu et al. 2011).

*S. cerevisiae* ORC recognizes specific DNA sequences within replication origins (Bell and Stillman 1992; Rao and Stillman 1995; Rowley et al. 1995; Dueber et al. 2007, 2011; Gaudier et al. 2007), while DNA structure and DNA-specific chromatin modifications appear more important for origin specification in metazoans (Remus et al. 2004; Eaton et al. 2011; Beck et al. 2012; Kuo et al. 2012; Cayrou et al. 2015). Interestingly, Cdc6 also consists of an N-terminal AAA^+^ domain and a C-terminal WHD, directly binds to ORC, and enhances the affinity and sequence specificity of budding yeast ORC (Speck et al. 2005; Speck and Stillman 2007).

However, the structural basis of origin recognition in eukaryotes was unknown for the longest time. Initially, crystal structures of archaeal ORC/DNA complexes revealed that both the β-hairpin wing and the helix–turn–helix (HTH) motif belonging to the WHD are deeply inserted into DNA grooves of the origin DNA. In addition, an initiator-specific motif (ISM) within the AAA^+^ domain contacts the DNA (Fig. 2A; Dueber et al. 2007; Gaudier et al. 2007). Both the ISM and WHD induce DNA bending, while the ISM also has a role in DNA sequence recognition (Dueber et al. 2007, 2011; Gaudier et al. 2007). Recently, the first high-resolution cryo-EM structure of DNA-bound ORC/Cdc6 in complex with Cdt1/MCM2–7 showed that *S. cerevisiae* Orc1–5 encircles DNA (Yuan et al. 2017). Here, the C-shaped structure of the Orc1–5 proteins is arranged in the order of Orc1–Orc4–Orc5–Orc3–Orc2, and Cdc6 fills the gap between Orc1 and Orc2, consistent with the previous low-resolution structure of *S. cerevisiae* ORC/Cdc6 (Chen et al. 2008) and the *D. melanogaster* ORC crystal structure (Fig. 2B; Bleichert et al. 2013). In this conformation, DNA makes multiple contacts with ORC/Cdc6 and is topologically trapped by the ring-shaped complex (Speck et al. 2005; Bleichert et al. 2015). Although Orc6 was not well resolved, a conserved C-terminal α helix was found to interact with Orc3 (Fig. 2B). Remarkably, this Orc6 helix is mutated in Meier-Gorlin syndrome. Indeed, this disease results in primordial dwarfism in humans, which is also caused by mutations in Orc1, Orc4, Cdt1, and Cdc6 (Bicknell et al. 2011; Tocilj et al. 2017). Consistently, in the case of Orc6, the mutation results in a defective Orc3 interaction, which in turn was shown to reduce MCM2–7 loading in *Drosophila* (Bleichert et al. 2013).

In the OCCM structure, the AAA^+^ domains and the WHDs of Orc1–5/Cdc6 form a central channel. In this context, Orc2, Orc4, and Cdc6 make direct DNA contacts, while Orc1, Orc3, and Orc5 do not touch the DNA (Fig. 2B; Yuan et al. 2017). Within the Orc2 protein, only the ISM of the AAA^+^ domain interacts with DNA, mostly contacting the phosphate backbone (Fig. 2C). Compared with most other species, budding yeast Orc4 contains an α-helix insertion, which is directly involved in DNA interaction. Unlike other Orc/Cdc6 proteins, Orc4 appears responsible for sequence-specific DNA interactions in the OCCM, as it uniquely makes base-specific contacts in the major groove of the DNA (Fig. 2D; Yuan et al. 2017). However, the functional relevance of these interactions is still outstanding. Moreover, site-specific DNA binding of the *S. pombe* ORC is also determined by the same Orc subunit (Chuang and Kelly 1999; Kong and DePamphilis 2001). Here, multiple AT-hook domains at the N terminus of *S. pombe* Orc4 are used for binding to replication origin sequences, highlighting the Orc4 protein as the most important module for sequence-specific DNA recognition in yeast. In contrast, *D. melanogaster* Orc4 and *H. sapiens* Orc4 are lacking the insertion α-helix and AT-hook domains. Intriguingly, metazoan replication origins share no common DNA sequence (Cayrou et al. 2015; Hyrien 2015). However, *H. sapiens* Orc1 has affinity for G-quadruplex ssDNA (Hoshina et al. 2013), shown to act as an origin-positioning motif (Valton et al. 2014), while DNA topology has been shown to be an important determinant for *Drosophila* ORC–DNA interactions (Remus et al. 2004). Moreover, epigenetic modifications, in particular H4K20 dimethylation and trimethylation, promote chromatin recruitment of *H. sapiens* ORC via an Orc1 bromo-adjacent homology (BAH) domain and DNA licensing (Beck et al. 2012; Kuo et al. 2012).

Besides *S. cerevisiae*, Cdc6 also plays a role in DNA binding in the OCCM (Yuan et al. 2017), with its ISM and WHD making prominent contacts with the phosphate backbone (Fig. 2A,C). Unlike archaeal ORC/Cdc6, *S. cerevisiae* Cdc6 uses only the WHD β-hairpin wing to contact DNA. Basically, *S. cerevisiae* Cdc6 contributes toward DNA binding at two levels: *S. cerevisiae* Cdc6 interaction with *S. cerevisiae* ORC traps DNA in the central channel, and, in addition, its two nucleic-acid binding surfaces contact the DNA directly, providing a structural explanation of why *S. cerevisiae* Cdc6 results in increased affinity of *S. cerevisiae* ORC for DNA (Mizushima et al. 2000). However, how the Cdc6 ATPase-dependent regulation of sequence specificity works (Speck and Stillman 2007) is still unknown at a structural level. The crystal structure of the *D. melanogaster* ORC core revealed that *D. melanogaster* Orc3–4–5 has a configuration similar to that of *S. cerevisiae* and *H. sapiens* Orc3–4–5, but the whole structure adopts an autoinhibited conformation that is incompatible with DNA and Cdc6 binding (Bleichert et al. 2015; Tocilj et al. 2017; Yuan et al. 2017). Aligning the *D. melanogaster* ORC structure to the *S. cerevisiae* ORC structure shows that the *D. melanogaster* Orc1 AAA^+^ domain and the *D. melanogaster* Orc2 WHD cover the central channel of the ORC DNA passage (Fig. 2E; Bleichert et al. 2015; Yuan et al. 2017). Thus, the conformational changes of those regions must be essential for DNA interaction and are also required for Cdc6 contacting Orc1 and Orc2 in *Drosophila*. With Orc1, Orc4, and Orc5 binding to ATP and ATP binding to Orc1 being essential for ORC/Cdc6/DNA complex formation (Weinreich et al. 1999; Gillespie et al. 2001; Klemm and Bell 2001; Speck et al. 2005; Randell et al. 2006; Speck and Stillman 2007), it appears possible that the interaction with ATP may result in strong conformational changes in the *D. melanogaster* Orc1 AAA^+^ domain and the *D. melanogaster* Orc2 WHD.

In summary, the recent structural insights reveal decreasing DNA sequence specificity during evolution. Archaeal ORC/Cdc6 proteins are forming multiple DNA sequence-specific contacts, and *S. cerevisiae* Orc/Cdc6 proteins are making fewer, mostly phosphate backbone and some base-specific contacts with DNA, while *H. sapiens* ORC is missing the sequence-specific DNA recognition motifs observed in yeast. The structural basis of topology-specific DNA recognition in *D. melanogaster* ORC and G-quadruplex-specific ssDNA binding in *H. sapiens* ORC is still outstanding and will offer further insights into the definition of replication origins in metazoan.

## General organization of MCM

**Figure 3.**
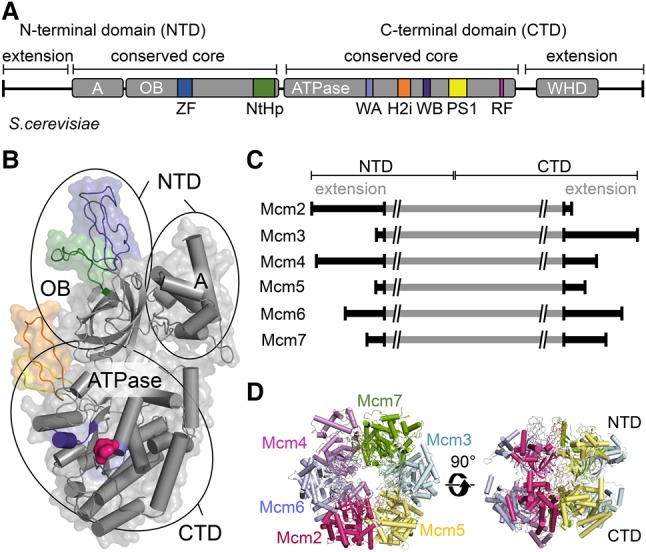
MCM2–7 helicase function arises from its architecture. (*A*) Domain structure of an Mcm protein with its four domains: A, oligonucleotide binding (OB), ATPase, and WHD. The color-coding for specific motifs—zinc finger (ZF), N-terminal hairpin (NtHp), Walker A (WA), Walker B (WB), presensor-1 (PS1), helix 2 insertion (H2i) β hairpin, arginine finger (RF), and WHD—is identical in *A* and *B*. (*B*) Atomic model of an exemplary Mcm subunit (PDB: 5U8S; Mcm2 of CMG bound to a replication fork) outlining its domain organization, with the WHD not shown. (*C*) Mcm subunits show different N-terminal and C-terminal extensions. (*D*) The MCM2–7 hexameric ring structure extracted from the CMG bound to a replication fork viewed from the top and side (PDB: 5U8S).

The MCM2–7 proteins are very well conserved from yeast to humans, while, in archaea, highly related homologs exist that assemble in homohexameric complexes. The six distinct subunits that make up MCM2–7 are dominated by a two-part domain structure (Fig. 3A–C): (1) the N-terminal protein interaction and DNA-binding domain and (2) the highly conserved C-terminal AAA^+^ ATPase motor domain (Fig. 3A,B; Costa and Onesti 2009). The two domains shape the characteristic dumbbell silhouette of the Mcm subunits, generating double ring structures in the context of the MCM2–7 hexamer (Fig. 3B,D).

The N-terminal domain (NTD) can be divided roughly into two subdomains. (1) α Helices in the regulatory subdomain A form a compact bundle on the outside of the helicase ring. This domain has been suggested to regulate MCM helicase activity and function as a conformational switch in archaea (Slaymaker and Chen 2012; Miller and Enemark 2015). Its role in eukaryotes is less clear. (2) The second subdomain exhibits an oligonucleotide-binding (OB)-fold. This fold is involved in making contacts with ssDNA, as seen in the context of *Pyrococcus furious* MCM, with a potential role in initial DNA unwinding during the DH activation (Froelich et al. 2014). In addition, this domain also contributes via hydrophobic interactions to oligomerization of MCM2–7 into the characteristic hexamer structure (Bochman and Schwacha 2009). The OB-fold is interrupted by a DNA-binding N-terminal hairpin (NtHp) and a zinc finger (ZF). One of the main functions of the ZFs is to form the DH interface, which gives rise to the extraordinary salt stability of the complex (Li et al. 2015; Zhai et al. 2017). N-terminal extensions (NTEs) (Fig. 3C) distinguish eukaryotic Mcms from their archaeal counterparts. The large NTEs in Mcm2, Mcm4, and Mcm6 are mostly disordered and regulate both initiation and fork progression in a DDK-dependent manner (Sheu and Stillman 2006, 2010; Randell et al. 2010; Sheu et al. 2014, 2016), while the Mcm2 NTE is also important for replication-coupled assembly of chromatin (Huang et al. 2015; Richet et al. 2015).

The C-terminal domain (CTD) of Mcm proteins contains the motor of the helicase that has several key features: the ATP-binding motif Walker A (WA) and ATP hydrolysis motifs Walker B (WB) and arginine finger (RF), with the latter being localized at each of the intersubunit interfaces. In addition, the CTD contributes to DNA binding using the DNA-binding presensor-1 (PS1) and helix 2 insertion (H2i) β-hairpin loops, which protrude into the inner channel. Furthermore, most of the Mcm proteins include beyond the ATPase domain C-terminal extensions (CTEs) (Fig. 3C), which are composed of WHDs and additional sequences representing protein interaction motifs with various functions during DNA replication (Li et al. 2015; Abid Ali et al. 2016; Yuan et al. 2016, 2017).

## MCM2–7 conformations in the OCCM, DH, and CMG reveal MCM2–7 ring-opening mechanisms

**Figure 4.**
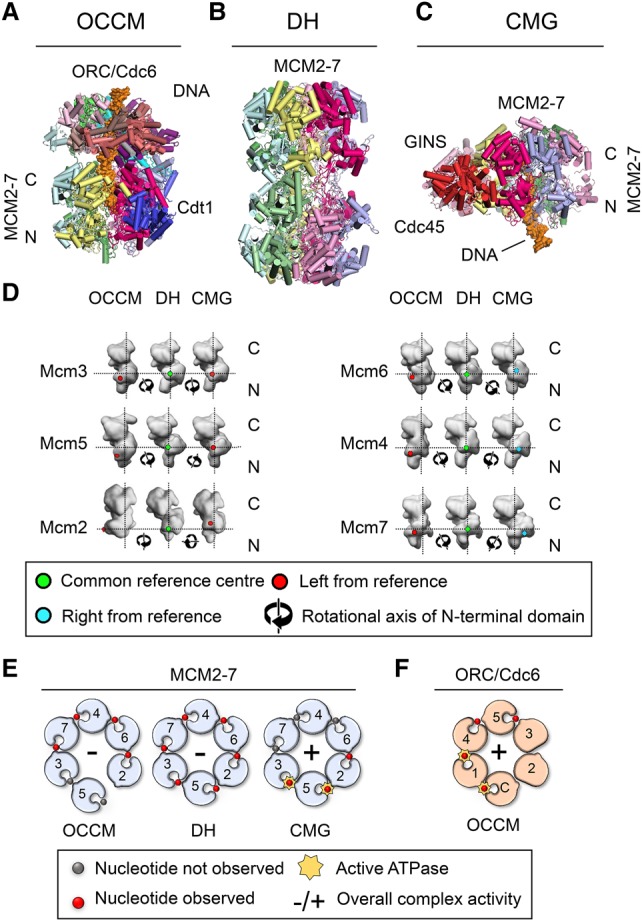
Structural changes in Mcm subunits dictate their function. Cartoon-style atomic models of OCCM (*A*; PDB: 5UDB), MCM2–7 DH (*B*; PDB: 3JA8), and CMG bound to a replication fork (*C*; PDB: 5U8S). (*D*) Comparison of the conformation of the MCM2–7 subunits in the OCCM, DH, and CMG. The CTDs of each Mcm subunit in the OCCM/DH/CMG conformations were aligned with the CTD of Mcm6 in the DH conformation, and this was taken as a fixed common position that was then used to generate the common reference center in the NTD (marked in green in the DH). This common reference point was used to detect the movement of the Mcm NTD in the OCCM and CMG relative to the DH, considering the Mcm NTD as a unit. Movement of the NTD toward the *left* is shown in red, and movement toward the *right* in is shown blue; the rotational axis relative to the common reference point is shown as a symbol. The alignment used the atomic structures of the proteins, but the figure depicts 10 Å surface view representations for improved clarity. (*E*) Schematic representation of the ATPase pockets of MCM2–7 in the OCCM, DH, and CMG. (*F*) ATPase pockets of ORC/Cdc6 in context of the OCCM.

Although MCM2–7 is at the core of the replication fork, the complex is unable to unwind or even associate with dsDNA on its own. Instead, MCM2–7 requires different sets of cofactors in order to carry out each of these reactions (Takeda et al. 2005; Evrin et al. 2009; Remus et al. 2009; Fernandez-Cid et al. 2013; Frigola et al. 2013). Indeed, MCM2–7 is known to form complexes with various factors during the different stages of DNA replication initiation and DNA synthesis (Fig. 1). Three of the complexes, representing prominent stages of DNA replication, have been structurally characterized recently by cryo-EM at high resolution: the OCCM, the MCM2–7 DH, and the CMG (Fig. 4A–C; Costa et al. 2011, 2014; Sun et al. 2013, 2014; Li et al. 2015; Yuan et al. 2016, 2017; Georgescu et al. 2017). Interestingly, the Mcm subunit conformations are different in these three complexes, reflecting distinct functional states. In order to visualize these differences, we performed a structural alignment of the CTDs of each Mcm subunit of the three complexes. Here we took advantage of a highly regular Mcm conformation observed across the six subunits in the MCM2–7 DH. Thus, the DH served as a unique reference point (Fig. 4D; labeled with a green dot) to understand the alternative Mcm conformations present in the OCCM and CMG. This analysis revealed that, in the OCCM, all MCM2–7 NTDs are left-twisted by various degrees, while, in the CMG, half of the hexamer has left-twisted NTDs (Mcm2/3/5), and the other half has right-twisted NTDs (Mcm4/6/7). In the following, we discuss the three different complexes and their functions in the context of these structural changes.

The OCCM complex represents a highly transient intermediate prior to ATP hydrolysis-driven MCM2–7 DH formation (Fig. 4A). In the OCCM, the helicase is associated with its DNA loader (ORC/Cdc6), and the dsDNA is already inside the central channel of MCM2–7, although the DNA entry gate between the Mcm2 and Mcm5 interface still remains partially open (Sun et al. 2013; Yuan et al. 2017). Crucially, while, in the MCM2–7/Cdt1 precursor, the six Mcm proteins are arranged in a spiral structure, in the OCCM complex, they are arranged in a plane similar to those in the MCM2–7 DH and CMG (Yuan et al. 2017; Zhai et al. 2017). Importantly, Cdt1, which is essential for ORC/Cdc6 and MCM2–7 association, interacts with both the core helicase and the DNA loader (Chen et al. 2007; Fernandez-Cid et al. 2013; Sun et al. 2013; Ticau et al. 2015; Yuan et al. 2017). Surprisingly, in the OCCM, the Mcm subunits exhibit a very different conformation compared with the DH or the CMG. Here, the Mcm2 NTD is dramatically left-twisted, which extends to the neighboring Mcm6 and Mcm4 subunits and a more minor left twist in Mcm3, Mcm5, and Mcm7 (Fig. 4D). This is likely due to the presence of Cdt1 and a network of interactions between the WHDs of MCM2–7 and ORC/Cdc6 (Yuan et al. 2017). The extended conformation allows Cdt1 to make multiple contacts with MCM2–7. Its N-terminal region is bound to the Mcm2 NTD, directly touching the twisted Mcm2 domain. A long loop links the Cdt1 N-terminal region to its C-terminal region. This C-terminal region itself interacts with Mcm6 but also with the Mcm4 NTD. Therefore, Cdt1 embraces half of the hexamer (Mcm2, Mcm4, and Mcm6). Interestingly, most of these Cdt1–MCM2–7 interactions have been also observed in the context of the Cdt1–MCM2–7 complex, with the exception of the highly conserved Cdt1–Mcm6 CTD interaction (Wei et al. 2010; Liu et al. 2012; Fernandez-Cid et al. 2013; Yuan et al. 2017; Zhai et al. 2017), suggesting that this interaction has an important role in OCCM formation and could contribute toward the structural changes in the Mcm NTDs (Fig. 4D). Intriguingly, it has been suggested that Cdt1 acts to stabilize Mcm2, Mcm4, and Mcm6, potentially allowing the movement of the other half (Mcm5, Mcm3, and Mcm7) (Yuan et al. 2017). Indeed, Cdt1 release from the OCCM is associated with MCM2–7 ring closure (Ticau et al. 2017). In sum, these data support the concept that Cdt1 is important for remodeling of MCM2–7 and closing of the twisted Cdt1/MCM2–7 ring during OCCM formation, but much more work is needed to directly link these events to Cdt1.

The MCM2–7 DH represents a very stable DNA replication intermediate. In this complex, the Mcm2/5 gate is tightly locked, and no DNA-unwinding activity can be detected until the DH becomes activated during the G1/S transition. Indeed, the two MCM2–7 hexamers are stacked via the NTDs in an interaction largely mediated by their ZFs. These tight interactions stabilize the two hexamers and hinder MCM2–7 ring opening (Sun et al. 2014). Moreover, in this head-to-head configuration, the interaction modes of each of the six ZFs are completely different, causing a visible tilt and twist between the two hexamers of the complex (Remus et al. 2009; Sun et al. 2014; Li et al. 2015). Although the functional relevance of the imperfect stacking of the two hexamers has not been demonstrated, it could have a role in the helicase activation process (Li et al. 2015). Interestingly, within the DH, the individual Mcm subunits are twisted between the NTD and CTD rings (Sun et al. 2014; Li et al. 2015). In fact, the NTDs of all six subunits display right-handed twists of varying degrees when compared with their conformation in the OCCM (Fig. 4D). This twist could represent an additional mechanism of sealing the weak Mcm2/5 interface (Samel et al. 2014; Sun et al. 2014). Furthermore, the distances between neighboring CTDs are different, with three tightly (Mcm4/7, Mcm7/3, and Mcm5/2) and three loosely (Mcm6/4, Mcm3/5, and Mcm2/6) packed pairs (Li et al. 2015). Again, the functional relevance is not known, but it could be possible that the loosely packed subunits could participate in MCM2–7 ring opening during helicase activation.

Finally, the CMG is the active form of the replicative helicase (Fig. 4C). This complex represents the center of the replication fork and actively unwinds dsDNA, exposing DNA single strands to the polymerases and allowing their replication (Langston et al. 2014). In this configuration, the leading strand passes through the MCM2–7 ring in a 3′–5′ direction, while the lagging strand is excluded from it, and the gate between Mcm2 and Mcm5 is sealed by tight interactions with GINS and Cdc45 (Costa et al. 2011; Georgescu et al. 2017). When the MCM2–7 DH becomes transformed into the CMG, the NTDs become reorganized (Fig. 4D). Here, the NTDs of half of the MCM2–7 ring (Mcm3, Mcm5, and Mcm2) are twisted to the left, while the NTDs in the other half (Mcm6, Mcm4, and Mcm7) are twisted to the right (Fig. 4D). This process involves a range of complex rearrangements in the three dimensions of space, and we propose that these structural changes reconfigure the central channel of the helicase to allow interaction with ssDNA.

## MCM2–7 ATP hydrolysis activities of the OCCM, DH, and CMG

MCM2–7 uses ATP binding and hydrolysis to unwind dsDNA during DNA replication. ATP hydrolysis is a coordinated process and requires the hexameric ring to be in a closed circular conformation (Schwacha and Bell 2001). The active ATPase sites are located within the Mcm dimer interfaces and form from conserved motifs of adjacent subunits; i.e., residues from both flanking protomers coordinate ATP binding and hydrolysis (Davey et al. 2003). The intersubunit nature of the active ATPase sites allows cooperative interactions that can be transmitted through the MCM2–7 ring. Indeed, incorporation of a single ATPase mutant into the MCM2–7 ring can result in a noticeable reduction in the ATP hydrolysis activity of the entire complex (Schwacha and Bell 2001; Davey et al. 2003; Ilves et al. 2010). However, some subunits, such as Mcm4, Mcm5, and Mcm7, appear to have a greater contribution toward the overall MCM2–7 ATP hydrolysis rate than others, as mutations in their WB motifs result in stronger ATP hydrolysis defects (Bochman et al. 2008; Bochman and Schwacha 2010; Bell and Botchan 2013). Many other ring-shaped hexameric motors show high cooperativity between active sites and sequential order of ATP hydrolysis (ATP hydrolysis in one subunit at the time following the order in the ring) (Abrahams et al. 1994; Liao et al. 2005; Adelman et al. 2006; Thomsen and Berger 2008; Eckert et al. 2012) but not all of them. For instance, studies on the hexameric protein unfoldase ClpX revealed that ATP hydrolysis in the hexamer is probabilistic. In ClpX, ATP hydrolysis in a given subunit depends on nucleotide binding of adjacent subunits, structural constraints, and substrate interactions but does not rely heavily on intersubunit cross-talk (Martin et al. 2005). Thus, ClpX ATP hydrolysis does not occur in the regular order of its six subunits but is fine-tuned for its specific function in unfolding proteins. In the context of MCM2–7, the data support a cooperative model of ATP hydrolysis. However, whether MCM2–7-dependent DNA unwinding at the replication fork follows a purely sequential order of ATP hydrolysis is still not clear.

MCM2–7 ATP hydrolysis during helicase loading has been analyzed by in vitro reconstituted reactions using a battery of ATP-binding and ATP hydrolysis mutants and a limited number of direct ATP hydrolysis assays (Fernandez-Cid et al. 2013; Coster et al. 2014; Kang et al. 2014). Here we discuss these data in the context of recent structural insights. Crucially, due to cryo-EM structures and fluorescence resonance energy transfer (FRET) data, we know that the MCM2–7 spiral becomes transformed into a nearly closed planar ring during OCCM formation, which then becomes further closed during OCM formation (Ticau et al. 2017; Yuan et al. 2017; Zhai et al. 2017). It is important to consider that the spiral-to-ring transformation introduces a tension into the complex, which puts the MCM2–7 complex under serious strain once it reaches the OCCM/OM/OCM stages. Consequently, mutations affecting the intersubunit interface geometry could block the ring closure, which in turn could affect (1) ATP hydrolysis-dependent Cdt1 release, (2) enclosure of DNA by the MCM2–7 ring, or (3) the ability of the complex to establish the correct hexamer–hexamer interface. Interestingly, MCM2–7 ATP-binding mutants are known to impair complex stability in the presence of low salt and elevated temperatures and display strong defects in the OCCM-to-DH transition (Coster et al. 2014; Kang et al. 2014). We suggest that the underlying reason for the pre-RC formation defect of Mcm ATP-binding mutants is the reduced complex stability, particular during the spiral-to-ring transition—a concept that could be tested using cryo-EM.

Furthermore, Mcm subunits are interconnected by RF mutants, which function for both complex stability and ATP hydrolysis (Coster et al. 2014). Two studies showed recently that mutation of the conserved Mcm arginine affects helicase loading, particularly when introduced in Mcm5 and Mcm6 but also in Mcm2, Mcm3, and Mcm7 (Coster et al. 2014; Kang et al. 2014). Moreover, arginine mutations in Mcm2 and Mcm5 specifically affect Cdt1 release. As Cdt1 release is a hallmark of pre-RC ATP hydrolysis, it was suggested that MCM2–7 ATP hydrolysis is essential for pre-RC formation. Interestingly, a different study showed that the RF mutation in Mcm3 does not have an impact on ATP hydrolysis rates during pre-RC formation (Fernandez-Cid et al. 2013). Direct ATP hydrolysis measurements for Mcm2 and Mcm5 RF mutants are still outstanding; therefore, it is not entirely clear whether these mutations affect only Mcm ATP hydrolysis (Coster et al. 2014; Kang et al. 2014) or may affect the intersubunit geometry and hence indirectly impair closing of the Mcm2–7 ring and/or Cdt1 release.

The cryo-EM analysis of the OCCM has provided further insights into the role of MCM2–7 ATP binding and hydrolysis during helicase loading. Here it was shown that ATP is at the interface of Mcm3/7, Mcm7/4, Mcm4/6, and Mcm6/2, where it possibly helps to stabilize intersubunit interactions. No nucleotide was detected at the Mcm3/5 or Mcm5/2 interfaces, consistent with the observation that the Mcm5/2 interface is broken (Yuan et al. 2017). Considering a cooperative MCM2–7 ATP hydrolysis model, the data would suggest that MCM2–7 ATPase activity is blocked or much reduced in the OCCM (Fig. 4E). However, Orc1 and Cdc6 are bound to ATP within the OCCM and appear primed for ATP hydrolysis (Fig. 4F; Yuan et al. 2017). Indeed, Cdc6 has been strongly implicated for ATP hydrolysis-dependent removal of failed helicase loading intermediates. Although the removal mechanism is not yet identified, Cdc6 ATPase activity is known to be induced during pre-RC formation (Fernandez-Cid et al. 2013), and Cdc6 ATPase mutants affect the release of Mcm3 (Coster et al. 2014), of MCM2–7 ATP-binding and ATP hydrolysis mutants that cause defective pre-RC formation (Coster et al. 2014; Kang et al. 2014), and of MCM2–7 loaded by Orc1–5 (missing Orc6) (Coster et al. 2014).

The role of Orc1 ATP hydrolysis during pre-RC formation is more complicated. An *S. cerevisiae* Orc4 RF mutant (Orc4R), which blocks ORC ATP hydrolysis (Bowers et al. 2004; Fernandez-Cid et al. 2013), showed no influence on ATP hydrolysis during pre-RC formation (Fernandez-Cid et al. 2013) or on MCM2–7 DH formation using a reconstituted pre-RC assay (Fernandez-Cid et al. 2013; Coster et al. 2014; Kang et al. 2014) but is lethal in vivo and allows only a single round of helicase loading using an extract-based pre-RC assay (Bowers et al. 2004). Furthermore, a Orc1 WB mutant (ORC-d1), which has defects in ATP binding and ATP hydrolysis (Klemm and Bell 2001), was analyzed under conditions of saturating ATP concentrations in order to test the role of Orc1 ATP hydrolysis during pre-RC formation. It was found that this mutant reduced helicase loading, Cdt1 release, and pre-RC-dependent ATP hydrolysis (Fernandez-Cid et al. 2013), suggesting that Orc1 ATP hydrolysis works in an ORC4R-independent manner during pre-RC formation. In summary, the role of ATP hydrolysis during pre-RC formation is complicated, as none of the mutants studied so far produces the same type of arrest in complex formation as has been observed with the slowly hydrolyzable ATP analog ATPγS.

As stated above, loading of multiple DHs at many replication origins is a way to store large amounts of the inactive replicative helicase before DNA synthesis takes place (Alver et al. 2014). Consistent with this notion, the DH is very stable even in the presence of high salt concentrations. Cryo-EM analysis revealed that ATP can be found at all of the Mcm subunit interfaces (Fig. 4E; Li et al. 2015). However, the strongly tilted conformation of MCM2–7 subunits resulted in structural changes in crucial ATP hydrolysis motifs, in particular the RFs localized at the intersubunit interfaces. Consistently, the DH was found to display minimal ATPase activity (Sun et al. 2014). Indeed, blocking MCM2–7 ATP hydrolysis represents a powerful mechanism to restrict MCM2–7 helicase activity in G1 phase, prior to its activation in S phase (Sun et al. 2015).

Progress has been made recently to better understand the fork organization (Abid Ali et al. 2016; Yuan et al. 2016; Georgescu et al. 2017). The very first cryo-EM structure of the *S. cerevisiae* CMG in complex with a forked DNA allowed Georgescu et al. (2017) to propose a new model of the replisome architecture, generating an improved framework to understand DNA synthesis. Most importantly, it was observed that, in the *S. cerevisiae* CMG, the NTD is at the leading edge of DNA unwinding (Georgescu et al. 2017). In contrast, a previous model suggested that the CTD is in front, which was based on FRET experiments with archaeal Mcm on a forked DNA and cryo-EM structures of the *D. melanogaster* CMG in complex with ssDNA (McGeoch et al. 2005; Costa et al. 2014). The new model, with the NTD near the fork, makes sense, as it places the leading and lagging strand polymerases in the correct locations for DNA synthesis, but additional studies would be useful to confirm these data and fully understand the DNA path through the replication fork (Sun et al. 2015; Miller and Costa 2017). In order to unwind the DNA, ssDNA needs to be actively propelled through the central channel of the CMG in a process that requires ATP hydrolysis (Moyer et al. 2006). Surprisingly, in *S. cerevisiae,* CMG ATP binding was detected at only three of the six Mcm interfaces; namely, Mcm3/5, Mcm5/2, and Mcm2/6 (Fig. 4E). Two of these (Mcm3/5 and Mcm5/2) correspond to the interfaces that are most important for helicase activity, as determined by analyzing the impact of ATPase mutants in the context of the *D. melanogaster* CMG (Ilves et al. 2010). Therefore, these studies suggest a differential contribution of the Mcm subunits toward the ATP hydrolysis and helicase activities of the CMG, with Mcm3, Mcm5, and Mcm2 being the key players. This suggests that MCM2–7 ATP hydrolysis does not follow the rotary model as observed in the E1 helicase or F1 ATPase (Enemark and Joshua-Tor 2008; Junge and Nelson 2015) but may have a substrate-specific activity similar to ClpX (Stinson et al. 2015).

## Evolutionary conservation of the MCM helicase DNA channel

**Figure 5.**
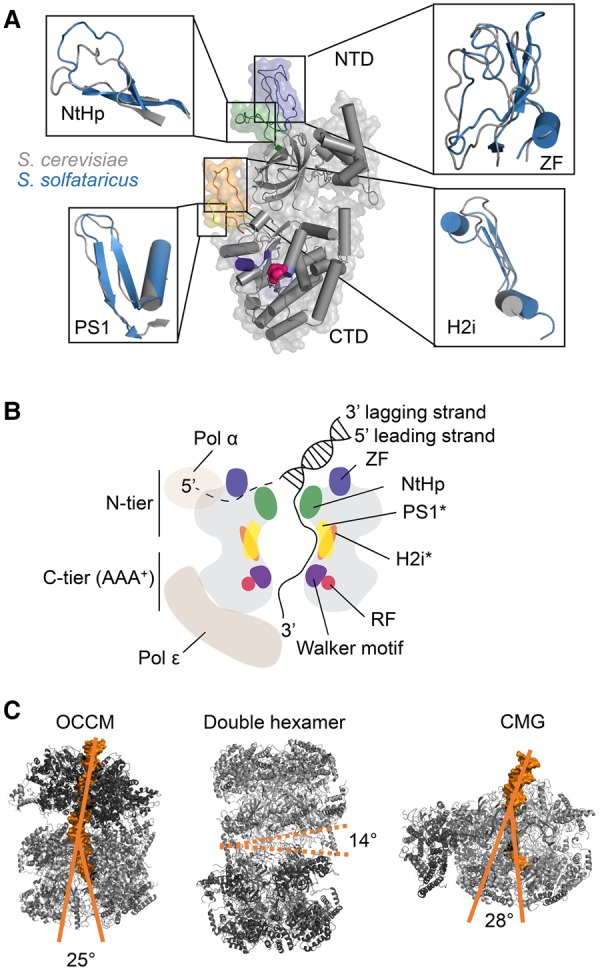
MCM2–7 DNA interactions within the OCCM, DH, and CMG complexes. (*A*) Detailed structural overlays of selected budding yeast (gray; PDB: 5U8S; Mcm2 of CMG bound to a replication fork) and *S. solfataricus* (blue; PDB: 3F9V) Mcm regions are shown. A superimposition of the PS1 loop, H2i loop, NtHp, and ZFs is depicted. (*B*) A schematic cut-through of the CMG with adjacent polymerases is shown with important domains labeled and color-coded as in *A*. The asterisk denotes that the hairpins of Mcm2/3/5/6 gather on one side of the central channel to interact with the passing ssDNA. (*C*) Structures of the budding yeast OCCM (PDB: 5UDB), DH (PDB: 3JA8), and CMG (PDB: 5U8S; CMG bound to a replication fork) are depicted. Furthermore, the central channels of the OCCM and CMG and its DNA bending are indicated. In the context of the DH, the interhexamer angle is shown.

The Mcms hydrolyze ATP to translocate on DNA, with the six subunits forming a strongly positively charged DNA channel (Kumar and Remus 2016). All Mcm proteins are conserved throughout evolution and are highly similar in their three-dimensional structure, especially in key residues that are necessary for DNA binding or translocation through the central channel (see Fig. 5A). Superimposition of published structures of Mcms from archaea (*Sulfolobus solfataricus*) and eukaryotes (*S. cerevisiae*) highlight their structural similarity (Brewster et al. 2008; Yuan et al. 2016). This is especially the case in regions such as the N-terminal ZF, PS1 loop, H2i loop, and NtHp (Fig. 5A), which are important for DNA binding and dsDNA unwinding. Indeed, archaeal Mcm mutants that lack either the PS1 loop or the H2i loop motif are still competent for binding DNA but are devoid of dsDNA-unwinding activity (McGeoch and Bell 2005; Jenkinson and Chong 2006). Although flexibility in these loops and structures may occur, their general structure and position within the helicase are very well conserved (Fig. 5A), suggesting a universal binding mode and DNA path. Even more, the structure of the archaeal Mcm PS1 loop is similar to the corresponding loops found in viral helicases, such as the E1 helicase or the SV40 T-antigen, hinting at a universal mechanism of DNA unwinding (Li et al. 2003; Abbate et al. 2004).

## Models for ORC-dependent MCM loading

Crucial MCM2–7 DNA interactions take place during ORC-dependent helicase loading, generating the critical topological link between the DNA and the helicase. Previous studies observed MCM2–7 in different conformations: The *S. cerevisiae* MCM2–7 hexamer was reported to be a closed ring, as observed by low-resolution negative-stained samples (Bochman and Schwacha 2007; Samel et al. 2014), which is also true for its archaeal and bacterial counterparts (Enemark and Joshua-Tor 2008; Miller et al. 2014). On the other hand, hexamers from *Drosophila* and the microsporidian parasite *Encephalitozoon cuniculi* were shown to adopt spiral-shaped or open-ringed conformations (Costa et al. 2011; Lyubimov et al. 2012). However, a recent study showed that *S. cerevisiae* MCM2–7 forms a left-handed spiral shape, with a 10–15 Å gap between Mcm2 and Mcm5 that is too narrow for DNA insertion (Zhai et al. 2017). In addition, it was found that the central channel of MCM2–7 was partially occluded by the CTEs of Mcm5 and Mcm6. This half-open complex is stabilized by Cdt1 binding to the N-terminal regions of Mcm2, Mcm4, and Mcm6 (Zhai et al. 2017). As MCM2–7 in this conformation does not allow DNA insertion, this suggests that the initial contact of Cdt1/MCM2–7 with ORC/Cdc6 needs to widen the Mcm2/5 gate to allow DNA entry. In the context of the OCCM, the MCM2–7 ring remains partially open (Sun et al. 2013; Yuan et al. 2017). The sealing of the Mcm2/5 gate requires ATP hydrolysis and removal of Cdt1 (Ticau et al. 2017), but the necessary structural rearrangements are still unknown. It will be fascinating to uncover the molecular mechanisms that allow controlled MCM2–7 ring opening, DNA insertion, and ring closure.

## A ssDNA path through the budding yeast MCM DNA channel

As discussed, the apparent DNA path through the *S. cerevisiae* CMG complex has been observed recently by using an artificial DNA fork substrate (Georgescu et al. 2017). Here, the *S. cerevisiae* MCM2–7 helicase travels in a 3′–5′ polarity with the N-terminal tier ahead of the C-terminal tier (Rothenberg et al. 2007; Costa et al. 2014; Sun et al. 2015; Georgescu et al. 2017). These data allow us to understand how DNA traverses through the CMG (Fig. 5B): The dsDNA enters the N-terminal tier of the helicase and is unwound. The investigators suggest that the lagging strand leaves the helicase sandwiched between the ZFs and the NtHps on the N-terminal surface of MCM2–7, where the polymerase α can directly prime lagging strand synthesis (Georgescu et al. 2017).

Upon strand separation, the leading strand is then passed through the positively charged central channel, where it interacts with the PS1 loop or the H2i loop of Mcm2/3/5/6 and adopts a right-handed spiral B-DNA form before being handed to polymerase ε for leading strand synthesis (Georgescu et al. 2017). Interestingly, earlier structural studies from archaea suggested that the ssDNA binds in the plane of the ring rather than perpendicular (Froelich et al. 2014). It will be interesting to investigate whether the new structural data of the CMG in complex with an artificial replication fork (Georgescu et al. 2017) will be supported by additional biochemical or in vivo data to verify the direction of DNA translocation of the CMG. Moreover, additional structures of replication fork intermediates captured in complex with DNA, which have been largely elusive so far, will be important to fully understand the path of dsDNA and ssDNA through the CMG.

## MCM and DNA channel flexibility during DNA replication

When comparing structures that depict different MCM2–7 states in DNA replication initiation in the order of their occurrence (OCCM → DH → CMG) (Fig. 5C), several differences are noticeable. Despite the overall unaffected structure of the two-tiered MCM2–7 helicase, internal rearrangements occur. In the OCCM, after the first MCM2–7 is loaded, DNA is bent by ∼25° at the ORC–MCM2–7 hexamer interface, which could represent a mechanism of DNA insertion (Yuan et al. 2017). Loading of the second hexamer and dissociation of the ORC leads to the MCM2–7 DH formation, in which hexamers are tilted by 14°, which may have a role in initial DNA unwinding (Li et al. 2015). Finally, dissociation of the DH and association of Cdc45 and GINS with the MCM2–7 rings lead to the assembly of the CMG. After activation, this complex continuously unwinds dsDNA to prepare it for replication. Through this, the dsDNA strand is bent again by ∼28° to the right of the vertical axis (Georgescu et al. 2017). This kink could be introduced by the necessity of efficiently unwinding the relatively rigid dsDNA. The designated lagging strand is proposed to leave the CMG on the surface of the Mcm3/Mcm5 interface (Georgescu et al. 2017).

## Protein interactions remodel MCM2–7 during different stages of DNA replication initiation

Unlike bacteria, eukaryotes coordinate their DNA synthesis with the cell cycle and organize their replisome around the replicative helicase (Stillman 2005). Clearly, the MCM2–7 proteins evolved significantly from their prokaryotic precursor and gained additional regulatory and functional features. Protein–protein interactions are at the core of this new functionality, and we discuss their roles in the context of MCM2–7 structure in this section.

## Two centers of the MCM2–7 interactome: the C-terminal and N-terminal protein-binding hubs

### C-terminal interactions

**Figure 6.**
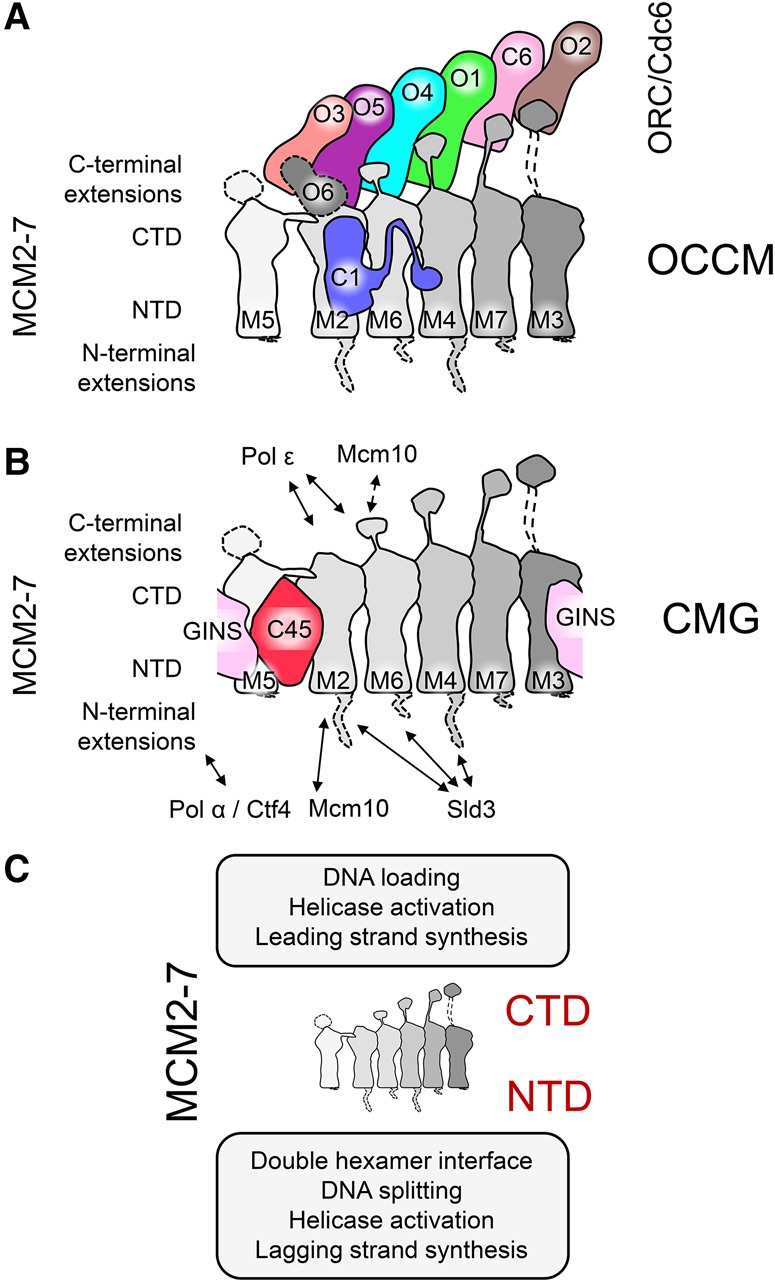
MCM2–7 protein–protein interactions in the context of OCCM and CMG. Interactions between the Mcm subunits (in gray gradient) and the Orc subunits (Orc1 in green, Orc2 in brown, Orc3 in salmon, Orc4 in cyan, Orc5 in purple, and Orc6 in gray), Cdc6 (light pink), and Cdt1 (blue) in the OCCM (*A*) and GINS (pale pink) and Cdc45 (red) in the CMG (*B*). The dashed lines indicate flexible regions not solved in the structures. (*C*) Summary of the different functions in which the MCM2–7 complex is involved though interactions with other proteins.

Cryo-EM structures of the OCCM complex (Sun et al. 2013; Yuan et al. 2017) and biochemical experiments (Fernandez-Cid et al. 2013; Frigola et al. 2013) have shown the important role of the Mcm C termini in pre-RC formation (Fig. 6A). In the following section, we discuss the progress in understanding the protein interaction interface of ORC/Cdc6 and Cdt1/MCM2–7. Indeed, Cdt1, which is essential for chromatin binding of MCM2–7 (Maiorano et al. 2000; Nishitani et al. 2000), interacts with the Mcm6 C terminus (Jee et al. 2010; Wei et al. 2010). Nuclear magnetic resonance (NMR) analysis of the human and budding yeast proteins identified the structure of this important interaction surface, highlighting the Mcm6 WHD for Cdt1 binding (Wei et al. 2010; Liu et al. 2012). Mutation of conserved amino acids in this domain affects the cell in two ways, blocking MCM2–7 nuclear import and DNA synthesis (Liu et al. 2012). In vitro analysis revealed that the Mcm6 WHD adopts an autoinhibited conformation that blocks the binding of MCM2–7 to ORC/Cdc6 (Fernandez-Cid et al. 2013). The structural basis of the Mcm6 WHD autoinhibition was observed recently (Yuan et al. 2017; Zhai et al. 2017). In the absence of Cdt1, the ORC/Cdc6 interaction with MCM2–7 is blocked due to a clash between Orc4 and the Mcm6 WHD. Cdt1 overcomes this block by reorganizing the Mcm6 WHD, which prevents the steric clash and enables tight ORC/Cdc6 interactions with Cdt1/MCM2–7. Other studies reported that the conserved C terminus of *S. cerevisiae* Mcm3 is also essential for the initial recruitment of Cdt1/MCM2–7 to ORC/Cdc6 (Frigola et al. 2013; Sun et al. 2013). Moreover, in the same studies, it was observed that Mcm3 interacts directly with Cdc6 and that the Mcm3 C terminus induces ORC/Cdc6 ATP hydrolysis with a role in quality control of complex assembly. Finally, the Mcm3 C-terminal region also contains a WHD that interacts with Cdc6 and Orc2, but the function of this interaction is currently unknown (Yuan et al. 2017).

In *S. cerevisiae* Mcm2, unlike the other Mcm subunits, the CTE is missing (Fig. 3C). Interestingly, Mcm2 in higher eukaryotes contains a C-terminal WHD. Furthermore, these eukaryotes also possess geminin, a crucial inhibitor of pre-RC formation (Lee et al. 2004). Interestingly, the N-terminal part of *H. sapiens* Cdt1 interacts with geminin; due to this proximity, geminin is well placed to interact with the Mcm2 CTE. We suggest that the Mcm2 WHD may have coevolved as an additional geminin-anchoring point and in this way could block pre-RC formation by interfering with direct Cdt1–MCM2–7 interactions.

Within the MCM2–7/Cdt1–ORC/Cdc6 interface (Yuan et al. 2017), the two complexes are tethered together using flexible links. These connections are made up from the Mcm CTEs and contain a flexible linker and a Mcm WHD, which latches onto ORC/Cdc6. In addition, the CTDs of Mcm2, Mcm6, and Mcm4 are connected directly to the WHDs of Orc5, Orc4, and Orc1, respectively, linking one half of the complex more tightly to ORC/Cdc6, while the other half remains more mobile. Although the functional relevance of these interactions is not yet entirely clear, this built-in flexibility has been suggested to be involved in the process of DNA loading (Yuan et al. 2017).

In contrast to the OCCM, the Mcm CTEs appear mostly flexible in the MCM2–7 DH (Li et al. 2015). However, in the CMG, the CTEs of Mcm4, Mcm5, and Mcm6 are more rigid. Two CMG cryo-EM structures—an Apo form and a variant bound to a fork DNA—have highlighted that the CTEs can adopt different conformations. Without DNA, the CTEs of Mcm5 and Mcm6 partially occlude the DNA channel, but, in the context of the replication fork, these are repositioned to allow ssDNA passage through the helicase, with the Mcm4 CTE touching the DNA itself, suggesting a complex mechanism of DNA sensing (Georgescu et al. 2017). In addition, the Mcm6 CTE makes contacts with Mcm10, but this is not essential for Mcm10 recruitment or DNA replication initiation (Fig. 6B; Douglas and Diffley 2016). At the C-terminal face, the CMG expels ssDNA from its central channel. Recently, polymerase ε was found to be localized in this position, contacting the CTDs of Mcm2 and Mcm6, ideally placed to carry out leading strand DNA synthesis (Fig. 6B; Sun et al. 2015). In summary, the MCM2–7 C-terminal face has a major role in MCM2–7 loading, helicase activation, and leading strand DNA synthesis (Fig. 6C).

### N-terminal interactions

During MCM2–7 DH formation, the N-terminal face of the two hexamers bind to each other, resulting in a highly stable complex. Within this complex, the long NTEs of Mcm2, Mcm4, and Mcm6 likely adopt a very flexible conformation, as they were not well resolved in the cryo-EM structure (Li et al. 2015). Once the cell enters S phase, DDK phosphorylates the MCM2–7 DH (Sun et al. 2014) to promote CMG formation and replication fork assembly. Specifically, the Mcm2, Mcm4, and Mcm6 NTEs are major sites for DDK phosphorylation and serve as binding sites for Sld3 (Fig. 6B; Sheu and Stillman 2006; 2010; Randell et al. 2010; Bruck and Kaplan 2015; Herrera et al. 2015; Deegan et al. 2016; Fang et al. 2016). In the CMG cryo-EM structures, the NTEs are only poorly resolved, again suggesting a flexible conformation (Yuan et al. 2016; Georgescu et al. 2017). However, upon interaction with specific partners, these flexible NTEs are likely to adopt a specific structure. In the case of Mcm2, a section of the NTE was crystallized together with an H3/H4 dimer and assumed a defined structure encircling the histones. Accordingly, this Mcm2 NTE has been suggested to play an important role in nucleosome recycling (Huang et al. 2015; Richet et al. 2015). Within the CMG, the NTDs are kept in a stable ring conformation through multiple interactions with Cdc45 and GINS. Here, Cdc45 interacts with the NTDs of Mcm2 and Mcm5 (Costa et al. 2011; Abid Ali et al. 2016; Simon et al. 2016). Cdc45 also closely interacts with the four proteins that constitute the GINS complex, which in turn binds the NTDs of Mcm3 and Mcm5. This broad system of interactions between GINS, Cdc45, Mcm2, Mcm3, and Mcm5 has been hypothesized to serve as a mechanism that keeps the Mcm2/5 gate in a closed state (Fig. 6B; Costa et al. 2011). Similarly, Cdt1 can contact Mcm2, Mcm6, and Mcm4 due to its striking extended conformation (Fig. 6A). However, this alternative network of interactions induced by Cdt1 has been suggested to have a different function, potentially allowing opening and closing the Mcm2/5 gate during OCCM formation (Ticau et al. 2017; Yuan et al. 2017; Zhai et al. 2017). Interestingly, although both Cdc45 and Cdt1 bind the Mcm2 NTD, they contact mutually exclusive surfaces of Mcm2, with Cdc45 binding the Mcm5-proximal part, while Cdt1 contacts the Mcm6-proximal section. Therefore, the two proteins might affect Mcm2 in alternative ways, with Cdc45 closing the gate to support helicase activity, and Cdt1 allowing opening and closing of the gate during MCM2–7 loading. Moreover, Mcm10 might affect these surfaces as well, as it contacts the Mcm2 NTD (Apger et al. 2010; Lee et al. 2010; Looke et al. 2017) in order to stabilize the CMG complex (Looke et al. 2017). At an in vitro assembled replication fork, ssDNA appears from the N-terminal MCM2–7 face during DNA unwinding (Georgescu et al. 2017). This ssDNA is well positioned to serve as a template for the lagging strand DNA synthesis. Indeed, DNA polymerase α/primase and Ctf4 were found to colocalize at the N-terminal MCM2–7 face in a low-resolution negative stain EM study (Fig. 3B; Sun et al. 2015). Ctf4 is a homotrimer that acts as a hub of protein interactions, linking several factors involved in DNA synthesis, chromatin remodelling, rDNA stability, DNA recombination, and sister chromatid cohesion to the CMG (Simon et al. 2014; Villa et al. 2016), while polymerase α primes DNA synthesis prior to extension by processive DNA polymerases (Zhang and O'Donnell 2016). These recent structural findings start to reveal a picture of the eukaryotic replisome assembly process as a whole; namely, the N-terminal MCM2–7 face has a major role in DH organization and stability, helicase activation, controlled opening and closing of the Mcm2/5 gate, and lagging strand DNA synthesis (Fig. 6C).

## Outlook

Structural biology has provided major biological insights into replisome assembly and function from the architectural point of view and also by providing detailed mechanistic insights. In the future, these data will stimulate the design of sophisticated biochemical and single-molecule experiments, facilitating the measurement of novel activities and revealing specific mechanisms and their dynamics. The replisome consists of 50 or more factors and many more associated factors; the integration of these into a structural and functional network will be an exciting task for the years to come. Translating this knowledge into the context of disease has already started (for example, in the case of Meier-Gorlin syndrome), but thousands of mutations, as observed in the context of cancer, have not yet been analyzed. Importantly, the structural and mechanistic insight into DNA replication initiation will help the development of novel inhibitors, which are aimed to block DNA replication before it even starts (Shreeram et al. 2002; Blow and Gillespie 2008). However, as the focus of drug development is considerably directed toward enzymes, it may still take some years before major progress is achieved.
